# Increased Left Ventricular Mass Index and Atrial Volume Index Are Associated with Atrial Fibrosis in Patients with Atrial Fibrillation

**DOI:** 10.3390/jcm14186432

**Published:** 2025-09-12

**Authors:** Simona Manole, Roxana Pintican, Claudia Budurea, Sorin Pop, Stefania D. Iancu, Loredana Popa, Mihaela Coman, Calin Schiau, Vasile Coman, Serban Schiau, Zoltán Bálint

**Affiliations:** 1 IMOGEN Research Institute, County Clinical Emergency Hospital, 400006 Cluj-Napoca, Romania; simona.manole@gmail.com (S.M.); zoltan.balint@ubbcluj.ro (Z.B.); 2 Department of Radiology and Medical Imaging, Faculty of Medicine, “Iuliu Hatieganu” University of Medicine and Pharmacy, 400012 Cluj-Napoca, Romania; 3 Faculty of Physics, Babeș-Bolyai University, 400084 Cluj-Napoca, Romania; 4 Institute of Life Sciences, University of Agricultural Sciences and Veterinary Medicine Cluj-Napoca, 400372 Cluj-Napoca, Romania; 5 Cardiology Department, “Emile Muller” Hospital, 68100 Mulhouse, France

**Keywords:** atrial fibrillation, echocardiography, cardiac MRI, atrial fibrosis associations, left atrial volume index, left ventricle mass index

## Abstract

**Objectives:** Our aim was to determine imaging-derived parameters from echocardiography associated with the presence of atrial fibrosis in a cohort of atrial fibrillation (AF) patients. **Methods:** Initially, 123 participants were included in this prospective cross-sectional observational study (clinicaltrials.gov: NCT03584126); after exclusion criteria, 112 full datasets were analyzed. All participants underwent clinical evaluation, echocardiography, and cardiac MRI. Overall, 29 patients with AF and left atrial (LA) fibrosis at MRI, 37 with AF and without LA fibrosis at MRI, and 46 healthy controls were included in the final database. Results: The cardiac structural parameters as assessed by MRI were not significantly different between AF patients with and without fibrosis, apart from LA volume. The area under the curve (AUC) reached a value of 0.69 when using body-surface-area-indexed LA volume (LAVi) determined by echocardiography as a factor associated with LA fibrosis in AF patients. Moreover, when detecting LA fibrosis using LAVi, an optimal cut-off value of 42.7 mL/m^2^ was obtained, resulting in 41.67% specificity and 88.46% sensitivity with a total accuracy of 65.06%. Testing BSA-indexed left ventricular mass (LVMi) as a factor associated with LA fibrosis, the optimal cut-off value was 140.2 g/m^2^, with 76.92% sensitivity, 58.33% specificity and 67.62% total accuracy for the discrimination between AF patients with and without LA fibrosis. A strong association between body-surface-area-indexed left atrial volume (LAVi) and the presence of atrial fibrillation was identified (54.5 mL/m^2^ vs. 29.8 mL/m^2^ in controls, *p*-value < 0.0001). **Conclusion:** LA volume indexed to BSA could be a promising tool for the identification of cardiac fibrosis in AF patients.

## 1. Introduction

Atrial fibrillation (AF), the most common cardiac arrhythmia, is one of the major causes of cardiovascular morbidity and mortality factors worldwide [1,2]. Left atrial (LA) structural remodeling is one of the pathogenic mechanisms involved in the occurrence and persistence of AF [3,4]. The degree of LA dilatation is highly associated with AF [5,6,7], which is a multifaceted condition that impacts not just the atria but also the entire circulatory system [8,9]. Thus, different diagnostic markers, such as LA structural and functional parameters, left ventricle (LV) diastolic function [10,11], CHA2DS2-VASc score [12], blood-derived biomarkers [6], mitral valve deceleration time combined with LA stiffness [13], and GLS [14] have been found to be relevant in the diagnosis of AF. Moreover, combinations of echocardiographic parameters were associated with AF presence, such as LV size changes combined with LA volume [15]. The combination of CHA2DS2-VASc score with AF burden [16], myocyte injury [12] or LA strain [17] has helped to define a stratification model for the risk assessment of AF patients.

One of the predominant structural changes in LA in patients with AF is the presence of fibrotic tissue, which can be quantified with late gadolinium-enhanced magnetic resonance imaging (MRI) [6]. Structural parameters of the heart and several pathological conditions, from which fibrosis quantification is feasible, are assessed mostly by cardiac MRI [18,19]. Echocardiography is more widely accessible, and part of a standard routine clinical cardiac evaluation, whereas cardiac MRI is sophisticated and requires greater patient compliance and more technical background [20,21]. The capacity of echocardiography to predict heart strokes, cardiovascular events or even to detect early stages of LA dysfunction has been reported previously [22]. The LA strain determined by echocardiography was used to detect LA fibrosis [23]. It is a useful parameter, but this is not yet routinely performed in clinical practice by cardiologists, as it is time-consuming. It is inversely related to LA wall fibrosis assessed by delayed-enhancement MRI, and these are related to the AF burden. Echocardiographic assessment of LA structural and functional remodeling is feasible and may be helpful in predicting outcomes in AF.

Histopathological studies of persistent AF have reported extracellular matrix remodeling with myocardial fibrotic infiltrations [24,25]. This may cause atrial dilation, and it may have a significant impact on wave propagation during AF [26,27]. Therefore, it is vital to find predictors of the presence of fibrosis in AF patients [28].

Several attempts have been made to identify blood-derived biomarkers of fibrosis in patients with AF (e.g., cardiac troponin I, N-terminal-pro brain natriuretic peptide, C-reactive protein, IL-6, BNP), but with varying accuracy levels [29,30]. Currently, various imaging techniques are used to determine the degree of LA dilation by measuring LA diameters and volumes (even indexed to body surface area—BSA), the latter being an important diagnostic and follow-up tool [31]. In addition, LV mass was identified as a predictor of thrombus formation in AF [32]. Therefore, a combination of imaging-derived parameters showing the presence of fibrosis in AF patients would be of great interest for patients and clinicians as well.

The aim of our study was to determine imaging parameters which reflect the presence of atrial fibrosis in a cohort of AF patients. In this pilot cohort study, a comparison of echocardiography-derived LA and LV parameters of patients with AF and healthy controls was performed, followed by an independent analysis for the comparison of AF patients with and without fibrosis on MRI.

## 2. Materials and Methods

**Clinical assessment:** Patients with AF and healthy volunteers underwent full cardiologic evaluation including electrocardiography (EKG) and echocardiography. Inclusion criteria were being 20–80 years old and 50–120 kg in weight. In the case of AF patients, the following criteria were mandatory: a diagnosis of persistent, permanent, or paroxysmal AF, constant medication for the last 2 weeks and optimal echocardiographic window. Regarding healthy volunteers, exclusion criteria were cardiovascular diseases, hypertension, or diabetes, whereas exclusion criteria for AF patients were other cardiac or chronic diseases known; patients under other chronic anti-inflammatory, oncological or study treatment; contraindication for cardiac MRI (prosthesis, pace-maker, metallic particles, pregnancy, known allergies); and altered renal function (creatinine clearance < 40 mL/min determined after Cockcroft–Gault) as presented in detail in [33].

Fibrosis was identified by MRI measurements, with the LA fibrosis group including patients with any detectable fibrosis in the left atrium. In addition to the demographic parameters, the CHA2DS2-VASc score [34] (C—congestive heart failure, H—hypertension, A—age ≥ 75 years, D—diabetes mellitus, S—prior stroke or thromboembolism, V—vascular disease, A—age 65–74 years, Sc—sex category) was determined and registered to assess the risk of stroke.

**Echocardiography:** A complete echocardiographic evaluation was performed with a Philips Affiniti 50 (software version 1.5.6.850) equipped with a Philips S4-2 Cardiac Sector Probe transducer. Data were stored and analyzed with QLAB Advanced Quantification Software (v10.5, Philips, Andover, MA, USA). From the apical four-chamber and two-chamber acoustic windows, two-dimensional images of the LA and LV were traced to calculate end-diastolic (EDV) and end-systolic volumes (ESV) using the ellipsoid model [35]. All previous values were normalized to BSA. Ejection fractions (EF) were calculated as well. The left ventricle parameters were measured automatically by the anatomical intelligence program from Philips based on 2D quantification (a2DQ), which determined the exact limit of the cardiac chambers from 2D echocardiographic images and calculated the volumes. Both LV and LA volumes were calculated by the area-length method. The diastolic function was assessed by pulsed-wave Doppler of the mitral and pulmonary venous flow velocities, tissue Doppler imaging of the septal, lateral, anterior, and inferior mitral annulus, and the average myocardial peak velocity of the early diastole (E′ peak), late diastole (A′ peak), and systole (S′ peak). TAPSE and myocardial strain were also determined. In the case of AF patients, after a pulse control, three successive cycles were chosen. Automated Cardiac Motion Quantification (aCMQ), a tool from QLAB focused on measuring cardiac tissue motion and strain, was used for global longitudinal strain. 

The determined parameters were LV EF, EDV, ESV, LA volume, global longitudinal strain (GLS), LV-S-lateral, LV-S-medial, mitral valve deceleration time (MitV-DecT), and LV mass. The LV mass was calculated using the formula

LV mass = 0.8 × (1.4 × (LVIDd + PWTd + IVSd)^3^ − [LVIDd]^3^)) + 0.6 (1)

All parameters used for LV mass calculation, left ventricular internal diameter end diastole (LVIDd), left ventricular posterior wall end diastole (PWTd) and interventricular septal end diastole (IVSd) were determined automatically. LA volume and LV mass were subsequently indexed to BSA (LAVi and LVMi, respectively), and used for further evaluation. Diagnostic cut-off values were adapted from the guidelines [35].

**Cardiac MRI**: Cardiac MRI measurements were performed within 10 days (mean of 4 days) of echocardiography and cardiac evaluation. All participants were imaged with a 3T whole-body MRI (3.0T Discovery MR750w General Electric) using the body coil for signal reception. Cardiac MRI required EKG gating and the breath-holding technique to overcome motion artifacts. The cardiac MRI protocol consisted of dark blood sequences (Black Blood SSFSE), FIESTA cine sequences (ALL FIESTA CINE AST—slice thickness 8, frequency 128, flip angle 60 and 20 views per segment) in short axis, two-chamber and four-chamber planes, and post-contrast sequences: rest perfusion (FGRE Time Course), angiography imaging (Aorta CEMRA Asset) and LGE/MDE images (2D MDE, 2D PSMDE-slice thickness 8, frequency 256, flip angle 20 and 24 views per segment) in short axis, four-chamber and two-chamber views. The MDE/LGE sequences were acquired with a time delay of 10 min after the injection of gadolinium (Gadovist, 1 mmol/mL, Bayer AG, Leverkusen, Germany). A 3D HEART sequence (slice thickness 0.8, frequency 256, flip angle 15) was also acquired and used to assess coronary abnormalities. The participants were constantly monitored and no immediate adverse effects were reported during the examination. 

The subjects fulfilling the previously presented inclusion criteria were considered suitable for cardiac MRI measurements. LA dimensions were measured at the end of the left ventricular systole (before mitral valve opening) when the left atrium reached maximum volume on FIESTA 2-CH and 4-CH cine images. LA volume was measured by the bi-planar area-length method with manually drawn endocardial contours in 2-CH and 4-CH views, with the exclusion of the left atrial appendage and pulmonary veins. The LA strain was not part of the imaging protocol in this study and was therefore not included in the analysis.

Indexed left atrial volume (LAVI) was measured at the televentricular systole, when the left atrium is at its maximum size. Gender differences were then accounted for by indexing the volume to body surface area using the Mosteller equation.

LA Volume = (8/3 π) × (A1 × A2/L) = 0.85 × (A1 × A2/L)(2)

LAVI = LA Volume/BSA(3)

Here,

- A1 = LA area in planimetric apical 4-chamber view (A4C);
- A2 = LA maximum planimetric area in apical 2-chamber view (A2C);
- L = Left artery long axis length (AL), determined as the perpendicular distance of the line measured from the mid-plane of the mitral annulus to the superior aspect of the AR.

The analysis for the quantification of LV volumes, myocardial mass, indexed mass and left ventricular ejection fraction (LVEF) was based on images with consecutive short-axis sections from the LV base to the apex in the end systole and end diastole using QMASS software 6.2 (Medis Medical Imaging, Leiden, The Netherlands) with automatic tracing of endocardial and epicardial contours of the left ventricular myocardium on all sections. Papillary muscles were considered part of the ventricular cavity. Left ventricular volumes and mass were normalized according to body surface area. Measurements were performed automatically and manually adjusted by consensus by two senior radiologists with 5 and 10 years, respectively, of experience in interpreting cardiac MRI examinations. Fibrosis identification was performed on short-axis 4-chamber and 2-chamber sections on sequences with injection of late Gadolinium enhancement (LGE) contrast agent.

**Figure 1:**
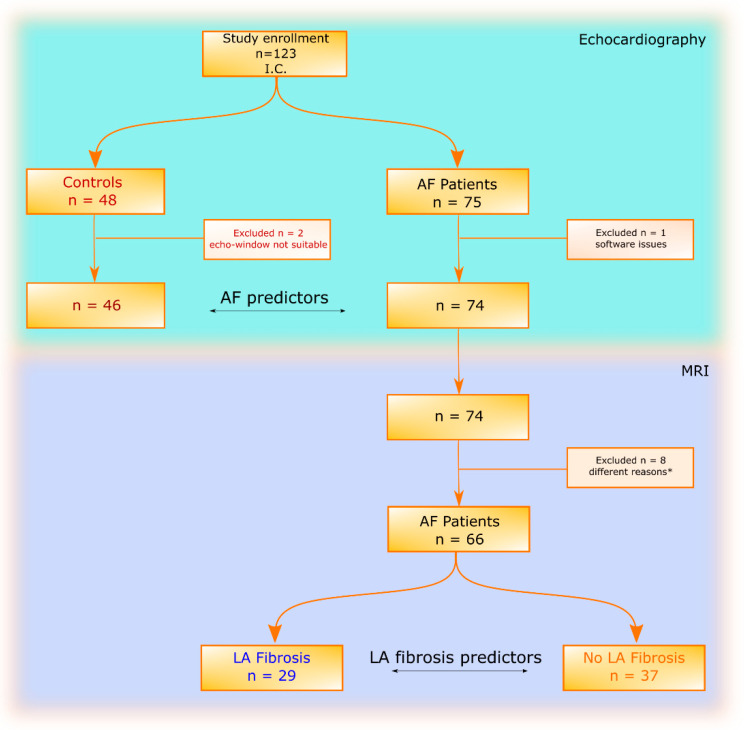
Flow chart presenting the study enrolment and the division of the dataset into two categories (46 controls and 74 AF patients) and two subcategories (29 AF patients with LA fibrosis and 37 AF patients without LA fibrosis) (* reasons described in the text).

**Statistical analysis:** The dataset was divided into two subgroups: healthy volunteers (control group) and patients with AF (AF patient group, Figure 1). The severity of AF was categorized by its duration (longer or shorter than 1 year). To investigate associations with LA fibrosis, the AF group was divided into two groups: Group 1 included patients with LA fibrosis, and Group 2 included patients without cardiac fibrosis or with cardiac fibrosis outside of the LA as determined by cardiac MRI. The normality of the distribution of all parameters within the groups was tested with the D’Agostino Pearson test. Data followed a non-gaussian distribution. Thus, they are presented as medians with interquartile range. Comparisons between the control and AF patient groups, as well as between patients with LA fibrosis and without LA fibrosis, were performed using an unpaired nonparametric *t*-test with Mann–Whitney corrections. The correlation between echocardiographic parameters was tested using nonparametric Spearman correlation. The statistical significance threshold for *p*-value was set to 0.05. Receiver operating curve (ROC) analysis was used to evaluate the performance of RM in identifying LA fibrosis based on LAVi and LVMi. We note that sensitivity and specificity from the confusion matrix were calculated at the chosen optimal cut-off, whereas ROC analysis summarized performance across all thresholds. Statistical analysis was carried out using GraphPad Prism Version 6.01 (GraphPad Software Inc., La Jolla, CA, USA).

## 3. Results

This observational study included 123 participants (Figure 1). Based on clinical assessment and echocardiographic examinations, the participants were divided into two groups: 48 healthy volunteers (control group) and 75 patients with AF (AF patient group). However, two healthy participants were excluded due to poor echocardiographic quality, and one AF patient was excluded due to software issues. After cardiologic evaluation with echocardiography, 74 AF patients were initially included, but 2 declined MRI procedures, 2 reported claustrophobia, 2 had metal implants, and 2 procedures could not be completed due to technical issues. Consequently, the final AF patient cohort comprised 66 complete datasets.

Based on the cardiac MRI examinations, these AF patients were grouped into 29 AF patients diagnosed with fibrosis in LA at MRI (LA fibrosis group), from which 21 had fibrosis only in the LA, whereas 8 had fibrosis in the LA and in other places in the myocardium; and 37 AF patients without LA fibrosis at MRI (no LA fibrosis group), out of which 29 had no cardiac fibrosis and 8 had fibrotic areas, but without fibrosis in the LA. To validate the cohort, we tested the association between echocardiographic parameters and AF presence, comparing AF patients and controls. The patient characteristics of the 74 AF patients (25 women and 49 men) and 46 controls (16 women and 30 men) included in the study are listed in Table 1. The ages of AF patients were significantly higher than those of controls, but there were no differences found in the BSA of the two groups. There was significantly increased LAVi, LVMi and ESV in AF patients as compared to controls, while EF, LV-S-med, LV-S-lat, and GLS had lower values in AF patients as compared to controls (*p* < 0.0001). In patients with AF, although LVMi was statistically significant, given that the AUC value = 0.66, the clinical utility was reduced. At the same time, LAVi proved to be a parameter closely related to atrial fibrosis, both clinically and statistically (*p* < 0.0001 associated with an AUC of 0.93) (Table 1).

Additionally, we used our cohort to further investigate the association of echocardiographic parameters with LA fibrosis in AF patients by comparing the values for AF patients with and without LA fibrosis at cardiac MRI.

Demographic data and echocardiographic parameters of patients with LA fibrosis at cardiac MRI (11 women and 18 men) and without LA fibrosis at MRI (12 women and 25 men) are presented in Table 2. When comparing AF patients with and without left atrial fibrosis on MRI, a longer AF duration in patients with LA fibrosis (65.5%) as compared to patients without LA fibrosis (54.05%) was present. Patients with LA fibrosis were significantly older (*p* = 0.020). The distribution of the CHA2DS2-VASc score values for the no LA fibrosis group was centered around 2, while in case of the LA fibrosis group the central score was 3, but no significant differences between the two groups were observed.

The association of cardiac structural parameters determined by MRI and LA fibrosis in AF patients was also tested. Significantly increased LA volume (*p* = 0.025), LA area 4C (*p* = 0.010) and LA longitudinal diameter (*p* = 0.020) were associated with LA fibrosis. No other parameter showed statistically significant differences between AF patients with and without LA fibrosis (Table 2).

**Figure 2:**
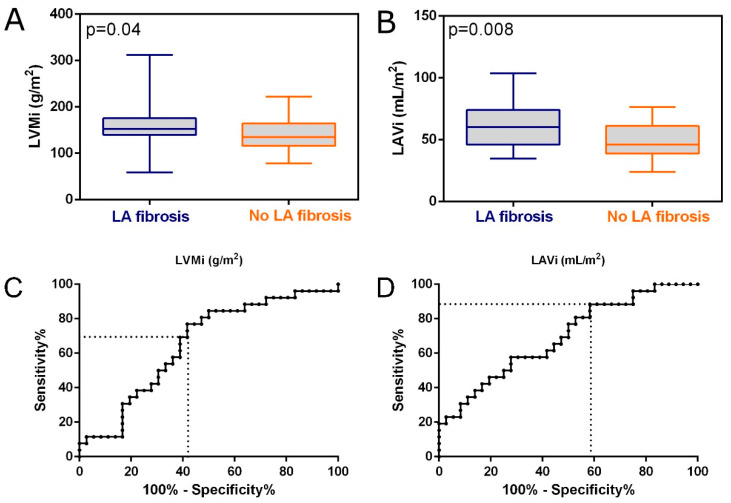
Box plots of (**A**) LVMi and (**B**) LAVi for LA fibrosis and no LA fibrosis groups. ROC curve for LA fibrosis and no LA fibrosis patients discriminated based on (**C**) LVMi with AUC = 0.65 and (**D**) LAVi with AUC = 0.69 (dotted lines indicate optimal cut-offs).

No differences between the LA fibrosis and no LA fibrosis groups were observed in echocardiographic parameters, such as EDV, ESV, MitV DecT, LV-S-med, LV-S-lat or GLS, or in diastolic function parameters, e.g., MitV E/A. However, LAVi was significantly higher for patients with LA fibrosis as compared to the no LA fibrosis group (60.2 mL/m^2^ with IQR 46.0–74.0 mL/m^2^ vs. 46.2 mL/m^2^ with IQR 38.8–61.1 mL/m^2^, *p* = 0.008). Another significant parameter for LA fibrosis discrimination was LVMi (*p* = 0.04), with a mean of 152.2 g/m^2^ with IQR 140.0–175.5 g/m^2^ in the case of the LA fibrosis group and 134.7 g/m^2^ with IQR 116.2–164.5 g/m^2^ in the case of the no LA fibrosis group (Figure 2A,B).

The two parameters (LVMi and LAVi) that showed significant differences between the LA fibrosis and no LA fibrosis groups were further used to test their power to diagnose fibrosis in LA. The model based on LVMi resulted in an area under the curve (AUC) of 0.65 (*p* = 0.041, Figure 2C), whereas in case of LAVi the AUC was 0.69 (*p* = 0.009, Figure 2D).

**Figure 3:**
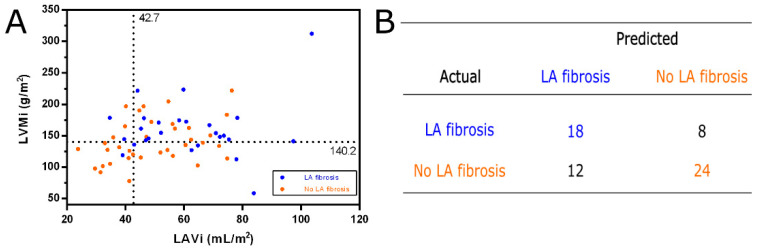
(**A**) LVMi distribution against LAVi for AF patients with the optimal cut-off for discrimination between LA fibrosis and no LA fibrosis. (**B**) Confusion matrix.

When detecting LA fibrosis using LAVi, an optimal cut-off value of 42.7 mL/m^2^ was obtained, resulting in 41.67% specificity and 88.46% sensitivity with a total accuracy of 65.06%. By using LVMi as an association with LA fibrosis, the optimal cut-off value was found to be 140.2 g/m^2^ with 76.92% sensitivity, 58.33% specificity and 67.62% total accuracy. We aimed to create a composite association with the combinations of LAVi and LVMi to identify patients with fibrosis in LA. Cut-off values of LAVi of 42.7 mL/m^2^ together with LVMi of 140.2 g/m^2^ had the strongest sensitivity for detecting LA fibrosis (Figure 3).

A multivariate analysis based on LAVi and LVMi with the specified cut-off values resulted in 24 patients with no LA fibrosis marked correctly (66.67% sensitivity), 18 patients with LA fibrosis as true positive (69.23% specificity), and 67.74% total accuracy (Figure 3B). Combining LAVi with LVMi significantly increased the total accuracy of the discrimination between AF patients with and without LA fibrosis from 65.06% in case of LAVi alone to 67.74% in the combined model. 

## 4. Discussion

Our study aimed to determine imaging parameters that can identify atrial fibrosis in a cohort of AF patients. We observed that while the structural changes in the left ventricle of AF patients were not influenced by the presence of fibrosis, patients with atrial fibrosis in our cohort exhibited significantly higher LVMi. This suggests a potentially effective combined model using LAVi and LVMi together to identify atrial fibrosis in AF patients. 

The presence of heterogeneously distributed fibrotic tissue in AF patients determined by the late gadolinium enhanced technique as a marker for AF recurrence after catheter ablation has been reported in the literature [36]. Although the relationship between fibrosis and AF is well documented, the implications of markers describing LV or mitral valves in the development of fibrosis is not fully understood [37]. Enlarged LA is associated with the development of fibrosis in LA. O’Neil et al. showed that people with lower LA volume and LAVi have a lower risk of developing atrial fibrosis and AF implicitly [38]. Moreover, the presence of fibrotic tissue in the LA wall was positively correlated with the increase in the volume of the LA [39]. Sivalokanathan et al. showed that AF patients with hypertrophic cardiomyopathy, which mainly affects the ventricles and interventricular septum, have a high burden of atrial fibrosis [40]. Even if the structural changes in LV in the case of AF patients were not affected by the presence of fibrosis, the LVMi was significantly higher in cases of patients with atrial fibrosis in our cohort. 

Regarding the pathophysiological mechanisms linking LVMi to atrial fibrosis, there are numerous studies that have documented and demonstrated that left ventricular hypertrophy (LVH) and left atrial (LA) fibrosis are interconnected, with LVH often preceding and contributing to LA fibrosis, which can lead to atrial fibrillation (AF) [41]. More specifically, an increased left ventricular mass index (LVMi) due to LVH can strain the left atrium, causing it to enlarge (LA enlargement) and develop fibrosis. This fibrosis can disrupt the electrical activity of the heart, increasing the risk of AF [42]. LVMi, a measurement of left ventricular mass relative to body size, is often used to assess the severity of LVH. Higher LVMi is associated with a greater risk of left atrial enlargement (LAE) and subsequent atrial fibrosis [41,43]. In addition to disturbed electrical activity, atrial myopathy is frequently responsible for an increased thromboembolic risk with ischemic events at multiple sites [44].

Echocardiography, especially advanced techniques like speckle tracking echocardiography used to calculate myocardial deformation parameters like the myocardial strain and the strain rate, has shown promise in detecting atrial fibrosis. However, its accuracy in predicting atrial fibrosis is generally considered lower than MRI. Sensitivities for echocardiography in detecting atrial fibrosis have been reported at around 30–70%, as obtained by our study. 

Generally, MRI is considered to be quite accurate in detecting atrial fibrosis, with reported sensitivities ranging from 71% to 97% and specificities ranging from 82% to 100%. These values can vary based on the study population and the criteria used for defining atrial fibrosis in the MRI images. However, echocardiography is generally more cost-effective and widely available than MRI. It does not require expensive equipment or extended scanning times, making it more accessible in various healthcare settings. Furthermore, echocardiography provides real-time imaging, allowing for the immediate assessment of cardiac function and structures during the examination. This can be particularly useful in dynamic situations such as stress echocardiography. While MRI is often considered more accurate for tissue characterization, these advantages make echocardiography a valuable tool, especially in settings where MRI may not be readily available or practical.

For a long time, atrial fibrillation was treated as a separate entity, but new studies demonstrate an interconnection between atrial fibrillation, atrial myopathy and thrombosis [43]. AF is the most common sustained cardiac arrhythmia in developed countries. On the other hand, atrial myopathy (AM) is a relatively new concept, consisting of structural and functional changes with remodeling of the atria, including fibrosis and changes in autonomic innervation that may lead to impaired atrial function and arrhythmia progression [45,46]. As Panteleimon et. al. have concluded in their research, AF and AM may be linked through their role in thrombogenesis and inflammation [43]. Further research and development of imaging techniques are needed, because while cardiac MRI and electroanatomical mapping detect and assess structural changes like fibrosis, it is also important to assess the degree of atrial remodeling [45].

A limitation of this study is the small number of patients, despite having enrolled a well-characterized patient population with the above indicated exclusion criteria to reduce the effect of other comorbidities or external factors upon the results. Moreover, no correlation between LAVi and LVMi and the type of atrial fibrillation could be found due to the small number of patients. A validation of these parameters could have been conducted in a longitudinal study, but the repeated cardiac MRI experiments were not indicated and were not feasible in our current clinical setting. One of the limitations of our study is that fibrosis assessment was binary (presence or absence), given that at the time of the study, in clinical practice, in our center, the cardiology team did not need the fibrosis percentage. The amount of atrial fibrosis did not influence the decision to ablate the patient or not, but of course it can orientate towards the ablative technique potentially necessary to obtain a favorable long-term result—pulmonary vein isolation, posterior box ablation (also known as posterior wall isolation PWI), Marshall vein alcoholization, etc. Quantification is necessary and will be the subject of a future study, where we will quantify and analyze fibrosis according to the UTAH classification [47]. As mentioned in the article written by Oakes et al. [47], DE-MRI of the LA is a noninvasive method useful for quantifying LA fibrosis, and is extremely important in determining the patient’s prognosis and the post-procedural recurrence rate after PVAI for AF. This idea is also supported by Verma et al. [48], who assessed the impact of left atrial fibrosis on the outcome of patients undergoing PVAI for AF; they concluded that atrial fibrosis is a powerful and independent predictor of procedural failure, being associated with a lower LVEF, larger LA size and increased inflammatory markers. The presence of LA fibrosis was only qualitatively assessed by the radiologists blinded to clinical characteristics and echocardiographic variables, indicating only the presence or absence of LA fibrosis. Although LA strain values were shown to correlate with LA fibrosis, another limitation of our study is that we could not access this parameter. Moreover, we did not obtain the diastolic function parameters for all patients enrolled in the study. A further large prospective cohort study of the identified combined parameters would be required to derive a clinically useful LA fibrosis prediction model regarding patients with AF.

Although the cross-sectional design does not permit an evaluation of predictive value for outcomes such as AF recurrence or stroke, our findings highlight parameters that warrant further investigation in prospective studies.

## 5. Conclusions

Body-surface-area normalized left atrial volume (LAVi) and left ventricular mass/BSA (LVMi) were identified as parameters associated with fibrosis in patients with atrial fibrillation. Combining LAVi and LVMi, we reached an accuracy of 67.74% for discriminating AF patients with LA fibrosis from those without LA fibrosis as determined by cardiac MRI. Cut-off values of LAVi of 42.7 mL/m^2^ together with LVMi of 140.2 g/m^2^ had the strongest sensitivity for detecting LA fibrosis. 

## Figures and Tables

**Table 1:** Demographic data and echocardiographic parameters for control and AF patient groups represented as medians (IQR) and the *p* values for the differences between control and AF patients group based on the Mann–Whitney test. The number of patients for each group is shown in brackets (* the parameter was not determined for all patients enrolled in the study).

	Control	AF Patients	*p* Value
No.	46	74	
Women/Men	16/30	25/49	
BSA (m^2^)	1.9 (1.8–2.1)	1.95 (1.85–2.1)	0.523
AF duration > 1 yr/<1 yr		43/31	
CHA_2_DS_2_-VASc score			
0/1/2/3/4/5/6/7/8/9/		3/19/21/19/5/3/1/0/0/0	
LAVi (mL/m^2^)	29.8 (25.3–34.7)	54.5 (42.2–69.6)	<0.0001
LVMi (g/m^2^)	128.5 (112.0–142.6)	144.5 (125.5–171.4)	0.006
EF (%)	62.8 (58.75–68.55)	56.7 (49.6–61.75)	<0.0001
EDV (mL)	95.9 (79.5–103.6)	90.1 (79.4–101.4)	0.427
ESV (mL)	35.6 (26.8–41.9)	38.4 (23.15–48.3)	0.043
MitV DecT (ms)	197.0 (173.0–232.0)	190.0 (158.0–229.8)	0.343
LV-S-med (cm/s)	7.9 (7.4–8.75)	6.3 (5.5–7.3)	<0.0001
LV-S-lat (cm/s)	9.2 (8.2–9.9)	7.45 (6.6–8.1)	<0.0001
GLS (%)	−21 (−23.0–19.0)	−16.0 (−20.0–15.0)	<0.0001
MitV E/A * (45/25)	1.20 (1.05–1.30)	1.55 (1.30–2.55)	0.0002
MitV E (mm/s) (45/70)	795 (667–888)	190 (150–229.8)	0.003
Pvein S (mm/s) (41/64)	582 (488–658.5)	416 (346.3–545.5)	<0.0001
Pvein D (mm/s) (39/64)	491 (432–568)	507.5 (426.3–710.3)	0.152
Pvein S/D (mm/s) (37/63)	1.2 (1.05–1.4)	0.8 (0.6–1.1)	<0.0001

**Table 2:** Demographic data and echocardiographic parameters for patients with LA fibrosis and no LA fibrosis, presented as medians (IQR), and the *p* values for the differences between the LA fibrosis and no LA fibrosis group based on a Mann–Whitney test. The number of patients for each group is shown in brackets (* the parameter was not determined for all patients enrolled in the study).

	LA Fibrosis	No LA Fibrosis	*p* Value
No.	29	37	
Women/Men	11/18	12/25	
Age	67 (61–71)	62 (53–68)	0.020
BSA (m^2^)	1.9 (1.8–2.1)	2.0 (1.9–2.1)	0.055
AF duration > 1 yr/<1 yr	19/10	20/17	
CHA_2_DS_2_-VASc score			
0/1/2/3/4/5/6/7/8/9/	2/6/5/10/4/2/0/0/0/0	1/12/14/7/1/1/1/0/0/0	n.s.
LAVi (mL/m^2^)	60.2 (46.0–74.0)	46.2 (38.8–61.1)	0.008
LVMi (g/m^2^)	152.2 (140.0–175.5)	134.7 (116.2–164.5)	0.041
EF (%)	57.35 (52.7–62.3)	58.0 (48.5–62.2)	0.970
EDV (mL)	83.35 (74.0–97.5)	95.2 (82.4–101.9)	0.105
ESV (mL)	33.9 (29.8–45.0)	39.8 (33.5–47.9)	0.137
MitV DecT (ms)	188.5 (163.3–212.8)	194.0 (161.5–243.0)	0.476
LV-S-med (cm/s)	6.3 (5.5–6.9)	6.3 (5.4–8.1)	0.527
LV-S-lat (cm/s)	7.45 (6.7–8.2)	7.45 (6.4–8.3)	0.640
GLS (%)	−16.0 (−20.0–15.0)	−16.0 (−20.0–−13.5)	0.962
MitV E/A * (11/12)	1.50 (1.30–2.60)	1.45 (1.22–3.07)	0.681
MitV E (mm/s) (27/36)	892 (805–1010)	870.5 (748.3–988.8)	0.855
Pvein S (mm/s) (27/31)	465 (347–582)	413 (348–519)	0.331
Pvein D (mm/s) (26/32)	492.5 (348.5–696.3)	578.5 (473.3–733)	0.152
Pvein S/D (mm/s) (25/32)	0.8 (0.65–1.3)	0.8 (0.525–1.1)	0.138

## Data Availability

The original contributions presented in this study are included in the article. For any further inquiries, please contact the corresponding author.

## Abbreviations

Left atrium—LA; left ventricle—LV; left atrial volume index—LAVi; left ventricular mass index—LVMi; atrial fibrillation—AF; body surface area—BSA; magnetic resonance imaging—MRI; end-diastolic volume—EDV; end-systolic volume—ESV; ejection fraction—EF; global longitudinal strain—GLS; left ventricular internal diameter end diastole—LVIDd; left ventricular posterior wall end diastole—PWTd; interventricular septal end diastole—IVSd; receiver operator curve—ROC; area under the curve—AUC.
